# It’s better together! European perspective on benefits and challenges associated with cross-border health communication campaigns

**DOI:** 10.1371/journal.pone.0204882

**Published:** 2018-10-17

**Authors:** Ricardo Rodrigues, Stefania Ilinca, Katharine Schulmann

**Affiliations:** European Centre for Social Welfare Policy and Research, Vienna, Austria; Universitat Luzern, SWITZERLAND

## Abstract

Recent years have witnessed greater involvement of European Union (EU) organisations in health communication campaigns that address chronic diseases and that are designed for implementation in multiple countries. This development raises challenges inherent in adapting the design of public health communication campaigns to multi-national settings. This article provides a first exploratory investigation of these challenges and how to address them based on data gathered from four expert focus groups, each concentrated on a common risk factor for chronic disease: smoking, alcohol consumption, unhealthy diet and sedentary lifestyle. Despite the exploratory nature of the data, it was possible to identify several common key challenges: variation in behaviours, social and cultural norms, and issues related to language and communication channels, the divide between EU stakeholders and local actors, and differences in national legislation and available resources. Two risk factor-specific challenges were also identified: effective messaging for complex issues (unhealthy diet) and the involvement of industry representatives (smoking, sedentary lifestyle). We propose conceiving of cross-national communication campaigns as providing a common blueprint and structure that can inform and support the development of differentiated yet harmonised local campaigns.

## Introduction

Accounting for more than 60% of all annual deaths worldwide, chronic diseases are the leading cause of mortality in the world and in Europe [1,2]. In the European Union (EU), 40% of the population aged over 15 live with one or more chronic diseases, resulting in reduced quality of life and considerable economic costs [3] While hereditary and environmental causes of chronic diseases are well documented, lifestyle factors and health behaviours also play a critical role [4–6]. It is estimated that by eliminating the main risk factors (tobacco use, unhealthy diet, physical inactivity and harmful consumption of alcohol) the prevalence of chronic diseases could be halved [7].

While health care policy is the responsibility of Member States, the EU plays an important role in allocating resources and coordinating efforts to address common challenges, such as portability of health care benefits (EU Directive 2011/24/EU), prescription standards, safety and quality standards, and prevention of communicable and non-communicable diseases [8]. Recognising that issues related to public health are not confined by national boundaries, the EU has held a mandate for public health going back to the Maastricht Treaty (1992). Through the first Community Health Strategy (2000) and the three subsequent Health Programmes, this mandate has expanded to include a focus on health promotion, education and information, and healthy lifestyles [9]. Implementation has often taken the form of consultations with key stakeholders as well as legislation and recommendations (e.g. the Strategy on Nutrition, Overweight, and Obesity in 2007; the Tobacco Products EU Directive 2011/24/EU, and the Action Plan on Youth Drinking and Childhood Obesity in 2014). In addition, the EU is responding to calls for increased coordination in risk communication and health promotion [10] by supporting a series of pan-European campaigns. Noteworthy are campaigns to promote tobacco free lifestyles and physical activity, including *HELP—For a life without tobacco*, *Ex-Smokers are Unstoppable*, the *European Week of Sport* and *BeActive*.

Recognising the increasing role of EU in spearheading communal action in the area of public health, the 2013 European Council senior level working group on “optimising the response to the challenges of chronic diseases”, called for integrated action across EU member states, including the implementation of cross-national health communication campaigns for primary prevention of chronic diseases [11]. We define cross-national health communication campaigns as cooperative efforts involving multiple EU Member States initiated at the supra-national, EU-level, and such campaigns are the focus of our analysis. These are distinct in their complexity from local cross-border cooperation, defined as collaborative efforts initiated by two or more bordering countries, which have a considerably longer tradition in Europe. To be sure, all health communication campaigns raise a host of complex challenges, but our focus here will fall on those that derive from the need for coordination and practice harmonization between numerous and often very different actors across borders.

Further research on cross-national campaigns is needed because many health challenges are taking an increasingly pan-European dimension and because coordinated cross-national efforts are associated with numerous benefits. These include reaching a broad target population; achieving economies of scale by pooling resources and coordinating efforts; promoting health communication in countries or regions where such strategies have traditionally been under-used [12]; ensuring an appropriate flow of resources into health promotion efforts [13]; facilitating learning and transfer of knowledge across borders and stakeholders [14]; and contributing to reduced health inequalities across countries [6,13]. Despite these advantages, research on cross-national health communication campaigns is scarce, with the specialised literature reporting mainly on the numerous instances of national and local level initiatives. A comprehensive literature review of communication campaigns addressing chronic diseases [15] found only one out of 63 selected articles [12] presented data on a European cross-national campaign. There is also scarcity of theoretical discussion of public health campaigns in cross-national settings. The available frameworks and conceptual models, understandably, are developed for the much more common case of local or national campaigns and focus on the details of messaging [16–18], targeting [19,20] and strategy [16,18] rather than on the complexities of managing heterogeneity of goals and target groups as well as cultural, institutional and political underpinnings between national settings. Our work aims to complement this body of research by focusing on the higher aggregation plane of cross-national campaigns where aspects related to heterogeneity, harmonization and coordination can, at times, be more salient than the practicalities of campaign design and implementation.

In order to enhance the effectiveness of cross-national health communication campaigns in Europe and in other trans-national settings, and facilitate capacity building in this area, it is important to systematise existing knowledge on coordinated public health interventions across several countries. We believe our research is timely as cross-national communication campaigns are an important and as yet under-utilised element within broader strategies to bring about structural changes in the behaviour of individuals. It also specifically contributes to address the above-mentioned call for greater coordination among EU member states for prevention of chronic conditions [11]. To this end we build on insights and experiences gathered from focus groups with diverse expert stakeholders to address two issues. First, the challenges specific to implementing communication campaigns in cross-national contexts and how best to address these; and second, the added value of cross-national health communication campaigns. We discuss results with a view to informing policy-making and contributing to the evidence base for the design and implementation of European cross-national health communication campaigns. It should be noted that while we believe the research presented here makes a meaningful contribution to the evidence base on this topic, the study carried out was exploratory in nature and our analysis is, owing to the scope of the overall project, based on limited data gathered over the course of the aforementioned focus groups.

## Data collection and methods

### Participants

The present study draws on data collected as part of a series of four expert focus groups, one held for each of the four main risk factors for chronic diseases: smoking, alcohol consumption, unhealthy diet, and sedentary lifestyle. Focus groups were chosen because they enable information to be gathered on a limited range of issues from a specialised group within a structured format [21]. Sampling was purposive in order to ensure a multi-disciplinary perspective [22] and was based on an extensive desk review of existing literature and conference proceedings as well as snowballing (i.e. requests for alternative recommendations) from contacted experts to identify participants with the desired expertise. As diversity in participant expertise is linked to increased range and depth of obtained information in focus group research, a priority in recruitment and selection was achieving a balanced mix of experts from different professional backgrounds (public health officials, academics/policy experts, communication and marketing experts, representatives of international and EU interest groups) [23]. Expertise working in multi-country communication campaigns addressing the risk factors under review was also an important criterion for participant selection. Finally, to ensure cultural sensitivity, recruitment attempted to gather a geographically diverse sample of participants. 117 experts were contacted, of which 32 accepted the invitation and 28 were ultimately able to take part in the focus group exercises (Table 1).

**Table 1:** Focus group attendance by professional background.

Expert background	Smoking	Alcohol consumption	Unhealthy diet	Sedentary lifestyle	All
**Public officials of Member States ^a^**	2	3	2	2	9
**Academics and policy experts ^b^**	-	1	2	3	6
**Communication and social marketing experts (i.e. researchers and practitioners with expertise in public communication)**	-	-	2	-	2
**Representatives of international organisations ^c^**	1	2	1	1	5
**European level interest groups ^d^**	3	2	-	1	6
**Total**	**6**	**8**	**7**	**7**	**28**

Disclaimer: Participants were invited on the basis of their expertise and their views may not necessarily reflect those of the institutions to which they are affiliated

^a^ Representative of local/regional health authorities in Italy and Spain; National Public Health Institutes in Italy, Hungary, Finland and the Netherlands; Public organizations for cancer prevention and research in Italy and Poland.

^b^ University Piedmonte Orientale, University of Trieste–IT; Birmingham University–UK; VU Amsterdam Medical Centre, TNO Netherlands, Wageningen University—NL; Aarhus University and University of Copenhagen–DK

^c^ WHO Europe; European Commission

^d^ Alcohol Youth Network; Eurocare; European Fetal Alcohol Spectrum Disorders Alliance (ESFAD); European Network for Workplace Health Promotion; International Sports and Culture Association; European Social Policy Network (ESPN); European Federation for Allergy and Airways Diseases Patients’ Associations (EFA); European Association of Communication Agencies

### Procedure

The focus group sessions were held in Brussels in February and March 2015 over the course of four days. The activities were designed to encourage consensus-building, allowing for the systematic collection of both individual and group generated insight. Each focus group was facilitated by a moderator and a note-taker and discussions were audio recorded following prior written consent. The structure of the exercises was harmonised across the four focus groups to ensure consistency and comparability. The first activity consisted of a short, open-ended brainstorming exercise in which participants worked in pairs to answer the question: “Drawing from your previous experiences in cross-national health communication, what are the main challenges associated specifically with cross-national campaigns?” Additional prompts included: “what is the added value of cross-national campaigns?”; “what are the structural cross-national challenges?”; “what specificities are associated with the risk factor?”; and “what is the role played by supra-national bodies?” Each pair of experts contributed between 3 and 5 challenges, recorded separately on index cards. The second activity consisted of a plenary clustering exercise in which each challenge was individually addressed and in which participants were asked to elaborate on each challenge. The moderator led and recorded the outcome of the discussion, clustering emerging themes on flipchart paper based on group consensus. Following the conclusion of each focus group, the moderator and note-taker held a debriefing session to discuss the key issues that emerged.

### Analysis

Given the limited research on cross-national communication campaigns, we followed an inductive approach to data analysis [24]. In a first step, the moderator and note-taker discussed and documented insights gained during the debriefing session [25]. The challenges recorded on index cards during the first activity were then analysed and coded. These codes emerging from the index cards formed the main themes (Table 2). Subsequently, the verbatim transcripts of each focus group were uploaded into MAXQDA software and analysed using thematic analysis. The coding of transcripts consisted of analysing each sentence or paragraph to understand ‘what is being said here?’ and thus attaching a code or label emerging from the data to blocks of text. Each code was then assigned to one of the main themes derived from the index cards or placed separately if it indicated a new emerging main theme. In the process we sought to identify common themes across the four data sets [26–28], to systematically appraise the codes and where appropriate, these were then clustered into more general categories or sub-themes [24]. The final code list included nine core themes and their respective sub-themes or codes (Table 2). In the final phase of analysis, all transcripts were re-coded according to the new code list and in-depth descriptions of the identified themes and sub-themes were extracted from the data. The frequency with which each sub-theme appeared or was coded across the four data sets is presented in Table 2.

**Table 2:** Main themes and sub-themes emerging from the focus group discussions, with frequency codes provided by risk factor.

	Main themes	Main sub-themes	Alcohol	Sedentary lifestyle	Smoking	Healthy diet
**Challenges common to all risk factors**	Heterogeneous epidemiological patterns	Different prevalence levels between countries	2	6	-	2
		Different behaviour patterns	9	-	-	6
		Variability in population groups at risk	-	1	8	1
	Different social and cultural norms	Cultural values and social norms	9	2	7	9
		Perceptions	3	2	4	-
	Variability in core communication tools and media	Adapting message to culture and target groups	14	9	15	19
		Choosing appropriate communication channel(s)	-	1	3	3
	Differences in institutional contexts and structural conditions	Differences in policies and policy priorities	1	6	-	-
		Differences in stakeholders/partners	-	4	-	1
		Variability in national legislation	5	3	7	1
		Differences in infrastructure	2	2	-	-
		Dynamic (historical) context	1	-	6	1
	Operational divide between supra-national and local actors	Identifying relevant local actors	3	4	9	2
		Subsidiarity	8	8	1	6
**Risk factor specific challenges**	Framing the message	Message complexity/controversy	8	-	-	7
	Involvement of industry in stakeholder networks	Scope and breadth of industry involvement	7	-	-	3
**Value of cross-national campaigns**	Supra-national bodies		3	6	3	4
	Mutual learning		2	6	-	2

To ensure reliability of the data analysis, two researchers coded each transcript independently. Any disagreements were resolved by the research team until consensus was reached. The final coding structure was discussed and agreed to by the research team. All transcriptions were anonymised prior to analysis.

## Results

We first present the challenges identified by focus group participants, presented in Table 2, which are classified into two main categories: challenges common or relevant to all groups; and risk factor-specific challenges. Each is presented in turn below with direct reference to their impact on the design and implementation of cross-national communication campaigns. We then present the findings that emerged from the focus groups concerning the added value of cross-national campaigns.

### Operational divide between supra-national and local actors

Participants pointed to the challenge inherent in the division of roles between the supra-national, national and local levels. They stressed that EU involvement is not necessarily seen as a positive in some countries, but rather as interference in national affairs. As a consequence, people may be less receptive to a campaign’s message if they perceive it to be an intrusion into their personal lives by an external, supra-national governmental body. On the other hand, the concept of subsidiarity, which in this context means decentralising responsibility for the planning and implementation of a campaign to the local level, is only a viable option if the national landscape is supportive and the local authority has the necessary capacity to lead the campaign. If it does not, due for example to lack of political will and/or lack of resources, then being part of a broader EU network was perceived to be a distinct advantage.

Participants across the focus groups agreed that epidemiological, cultural and institutional differences result in the need to identify relevant actors who are familiar with the local context. Strong local networks were considered key to the successful implementation of a cross-national communication campaign, as the key actors, people in the community who have authority and the trust of the population, e.g. medical doctors, vary from one local context to the next. Nonetheless, the use of local partners has its own challenges, because identifying and coordinating relevant stakeholders at different governance levels who represent a range of interests, was perceived as a considerable undertaking.

### Varying health behaviours, cultural and social norms

A major obstacle in designing cross-national health communication campaigns emphasized by nearly all participants is the marked differences in the patterns and prevalence of health behaviours in European countries. Participants emphasised that some countries are ahead of the curve in the “smoking epidemic” cycle (e.g. UK, Nordic countries), while others still face high prevalence and low rates of decline (e.g. Eastern and Central Europe). Dissimilar patterns are also observed for dietary habits, levels of physical activity and alcohol consumption. According to participants, such variability renders the definition of precise campaign objectives exceedingly difficult in a cross-national setting. The dynamic nature of health behaviour patterns at the national level also emerged, interwoven as they are with historical processes that include past legislation, policies and health campaigns, and shifts in cultural paradigms:

*“If you look at the percentage of people smoking which are more or less my age it’s lower*, *but among the young [smoking] is increasing again*. *So that means that the campaign*, *I mean the process of what we did before*, *was working very well*, *[*…*] but now we are missing the young*.*”* (Smoking #1, European-level interest group)

Differences in patterns and prevalence of health behaviours derive in part from contrasting cultural and social norms. This challenge was more prominent in the alcohol and unhealthy diet focus groups, as social norms were perceived to underpin the “drinking culture” and food consumption in each country. In the case of unhealthy diet, food consumption is also heavily determined by local availability of products. As a result, certain behaviours may have different underlying causes and require a differentiated approach within a cross-national communication campaign:

*“[*…*] for example*, *in Finland or in several other countries that have more salty fish—you know*, *this salty smoked fish— [*…*] this was chosen as the first target to decrease salt content*. *In Hungary it was not interesting because we don’t have such a product and we don’t eat that*.*”* (Unhealthy diet #9, Public official of Member State)

Social norms influence what is considered ‘healthy’, acceptable social behaviour, and the receptiveness to governmental intervention. For this reason, participants maintained that understanding variations in social norms is critical if a campaign is to be on target and on message. For example, food consumption is heavily determined by ingrained culinary traditions. With alcohol consumption, where evidence of its harmfulness is less absolute, and where the type of alcohol consumed differs between countries and population groups (e.g. spirits versus wine or beer), participants stressed that it can be difficult to decide between promoting adherence to general consumption guidelines, or focusing on, for example, heavy drinkers.

### Language differences and varying utilisation of communication channels and media

Social and cultural norms also inform language and messaging. Participants in all focus groups expressed reservations about the feasibility of having a European-wide message that both appeals to audiences across countries and is not so generic as to lose its impact and meaning. The exception to this was the above-mentioned tobacco cessation messages. On the other hand, tailoring a campaign message for each respective country context was seen as a costly endeavour as it requires more than mere literal translation. In order to effectively convey the campaign message to the desired target group, participants reflected that it is often necessary to adapt terminology, tone, expressions and even the role models used to deliver the message in order to capture local idiosyncrasies and to appeal to different attitudes and cultural values:

“…*due to cultural differences*, *you think that you [are] using the same words*, *but you could have completely different interpretations of that word (*…*) Or a referee [i*.*e*. *a role model] in one country could be seen as a big authority and in another country a big loser*.*”* (Sedentary lifestyle #5, Public official of Member State)

Users of specific types of media often belong to different socio-economic and demographic groups depending on the country or region, making it difficult to select just one channel of communication to suit every context. The participants in the smoking and sedentary lifestyle groups raised the issue of participation in social media to illustrate this point. Use of social media varies across Europe (common in Western Europe but displaying a steep age and socio-economic gradient in Eastern countries), as does Internet coverage and use of mobile technology. Experts indicated that more traditional communication channels including print media, television and radio should not be underestimated because in many contexts, these still have broad reach.

*“*…*in Denmark*, *for example*, *we have something called weekly sales flyers*. *All the big retail chains once a week send the printed materials to all households*. *That’s a very important channel in Denmark that most other countries don’t have*.*”* (Unhealthy diet #2, Academic and policy expert)

The importance of balancing the local cost of utilising a specific media channel with its expected impact was also emphasized. Given that both these dimensions are highly context specific, the advantage of formulating a uniform communication strategy at the cross-national level was strongly contested.

### Differences in institutional contexts and structural conditions

While communication campaigns are important, in order to achieve behaviour change participants stressed that campaigns must be integrated with wider policies and other support measures (e.g. counselling and treatment for smoking cessation, taxation policies). The challenge here is that the substance and scope of such policies and services vary substantially across countries. In fact, participants in three of the focus groups discussed the additional challenge posed by differences in relevant national legislation, which can affect the approach and messaging strategy of a campaign. Differences in tobacco and alcohol legislation governing taxation, marketing and distribution determine to a large extent the accessibility and societal perception of such products. Similarly, regulation and enforcement of product labelling and distribution standards for food products varies despite efforts for harmonisation across the EU.

Participants in all focus groups agreed that it is not only about current policies or infrastructure, but also about past national policies. In countries with longer histories of public health interventions and/or in which support measures have been in place for longer, there is a higher chance that communication campaigns will achieve their goal, not least because the infrastructure to carry them out is well-established.

*“*…*people are more aware in some countries*, *like in the UK they have a long history of public health campaigns and everybody is aware of [the] issues*. *That’s not the case*, *for example*, *in Eastern Europe*.*”* (Unhealthy diet #2, Academic and policy expert)

This speaks to the dynamic historical process alluded to before which shapes existing national regulatory frameworks and policies.

The dissimilar institutional and structural conditions prompted participants to question how cross-national health campaigns should prioritise the allocation of scarce resources in order to maximise the return on investment of public funds. While some countries might have the infrastructure in place to work towards the goals of a health campaign–thus enhancing its potential to change behaviour–in others this infrastructure might need to be built, requiring additional resources. The ability of cross-national communication campaigns to build capacity may thus not only be aligned with achieving behavioural change with the least resources spent.

### Framing the message

While the challenges described until now apply to all risk factors, two additional challenges emerged that are risk factor specific. The first is the complexity of messaging, which participants associated particularly with communication campaigns targeting unhealthy diets. In this area, campaign messages usually aim for behavioural convergence with certain nutritional standards, which were recognised to be both complex and disputed, as the evidence base linking specific dietary habits and health is subject to regular contestation by experts. Participants stressed that this renders the definition of a single comprehensive campaign message across countries effectively impossible. If one considers differences in national nutrition guidelines, the availability of certain foods and their relevance in the traditional diet and local food industry in each individual country, defining a ‘healthy diet’ in a consistent manner across the EU becomes a formidable challenge:

*“We know more or less from a nutrient perspective we have a good idea of the balance of nutrients*, *and we also have a good idea about food groups and what relative contribution they should make to a healthy diet*. *[*…*] But when it comes down to individual foods then that is very culturally specific*. *You can’t say in Nordic regions generally use olive oil because it’s not culturally applicable*, *it’s not available*, *it’s probably not that affordable*.*”* (Unhealthy diet #7, Representative of international organization)

Although less prominent, similar issues were raised in the sedentary lifestyle and alcohol consumption focus groups and are liable to affect all campaigns that attempt to address highly complex behaviours.

### Involvement of industry as partners

Whether or not, and if so to what extent, representatives of industry should be involved in cross-national communication campaigns constitutes the other risk factor-specific challenge. Participants in the smoking group dismissed the inclusion of industry as a partner. Those in the unhealthy diet group, however, indicated that as the availability and advertisement of healthier nutritional alternatives is crucial to behaviour change, the food production and distribution industries are important partners in any campaign coalition. These same experts nonetheless recognised that industry representatives can be reluctant to support such communication campaigns.

In the alcohol consumption focus group experts conceded that there are benefits to be gained from including responsible consumption messages in marketing campaigns developed by alcohol producers. Despite this, participants were keen to stress that “*We have to distinguish between partners and stakeholders*” (Alcohol #5, Public official of Member State), with the industry clearly identified as the latter. A similar discussion can be extended to the benefits of involving political stakeholders in the campaign coalition.

### Value of cross-national health campaigns

It was also clear from the participants’ accounts that while complexity in cross-national campaigns is inevitably linked to added difficulties, none of the challenges listed above is inherently insurmountable. We therefore settled on the terminology of challenge (implying a difficulty to be addressed) rather than drawbacks or limitations (more suggestive of a negative aspect that cannot be changed and must be accepted as such), as participants across the four focus groups also emphasised the advantages or value of cross-national communication campaigns. They referred to three main points. First is the ability of supra-national bodies such as the EU to bring together national institutions and to coordinate health campaigns built around common objectives or shared messages. While such common objectives seemed feasible in the context of smoking and sedentary lifestyle, there was scepticism about how realistic this would be for unhealthy diet and alcohol consumption. Secondly, cross-national health campaigns were perceived to have the potential to act as disseminators of good practice and capacity-building in national settings where expertise or policy relevance of health campaigns lags behind. Participating in a cross-national campaign may be seen as taking part in “*something bigger than just my country”* (Sedentary lifestyle #2, European-level interest group). According to participants, the institutional weight of the EU and accompanying resources can make a difference in such contexts. Finally, according to participants, a supra-national body operates with a longer-term perspective and can thus ensure that the health communication campaign is less dependent on national political cycles, particularly over longer implementation periods.

The value of cross-border public health communication campaigns boils down to ensuring goal and message coordination and capacity building, with a broader perspective and longer-term stability than can be routinely expected at the national level. In fact, the stable coordination and collaboration framework that cross-border campaigns provide goes much beyond the depth of providing a EU level recommendation for member states to individually plan and implement communication campaigns in isolation (or even local cooperation). Therefore, we argue the two approaches should be considered complementary rather than substitutes and should continue to be employed in parallel.

## Discussion

The purpose of this study was to identify the challenges specific to cross-national health communication campaigns within the EU community. Indeed, while the findings from the expert focus groups highlighted several challenges inherent to cross-national campaigns, the discussions also reinforced the fundamental value of such campaigns in the broader fight against chronic diseases. The main challenges raised by participants largely stem from the different epidemiological, cultural, and institutional realities that prevail in a given context, whether at the national or sub-national level.

In this section we elaborate the challenges identified in the results section above and propose a set of recommendations for overcoming these hurdles. Elsewhere, a series of key design principles for cross-border health communication campaigns has been proposed (see S1 Material), which reinterpret these challenges and formulate guiding principles to be applied in the design and roll out phases of these campaigns [29].

### The right message for the right people

In order to effectively reach its target audience, cross-national communication campaigns must be sensitive to variations in population health status and behaviour. This means carrying out basic demographic research during the planning phase and being strategic in selecting the target group(s). An understanding of the reasons behind demographic divergences is also important as regional and country health patterns continue to evolve and are closely linked to socio-political traditions [30,31].

Equally important is the need to consider cultural and social dimensions. If social pressure against smoking and alcohol consumption is high, a campaign addressing these risk factors can be more effective by highlighting the negative social perception rather than the adverse health impact [32]. In settings where these risky behaviours are more widely accepted, communication campaigns could raise awareness of the health risks through negative messaging.

### Working within countries’ political, policy and institutional frameworks

The level of commitment on the part of national policymakers to support a campaign is a particularly intractable issue. Political support for a campaign can wane with changes in government or in national priorities. The international nature of cross-national campaigns can serve to de-politicise a campaign and maintain a continuum of support irrespective of changes in the political environment. According to some participating experts, the strategy of designating an international organisation to act as an intermediary and as a more neutral face of a campaign can serve to ease potential frictions between EU and national actors. An additional advantage of this approach is that such organisations, such as the European Heart Network, have affiliate national branches whose networks are a natural starting point for building a coalition of stakeholders at the national and sub-national levels.

More important still, existing evidence suggests that health communication campaigns can be effective in raising awareness but their impact on actual behaviour change is limited unless they are coupled with complementary policies promoting structural change [33–35]. The EU, through its regulations, directives, and recommendations has some power to implement structural change and has done so in the past with a degree of success. A good example of this is the prohibition of smoking in public spaces [8]. However, as different legislative and infrastructural realities persist in different countries, developers of cross-national campaigns are advised to consider what can realistically be achieved by campaigns that are not tied to policies targeting structural change, and to modify the goals of a campaign accordingly. A noteworthy example is the lack of legislative and conceptual standardisation of what is considered to be a ‘healthy diet’ [36]. Experts in the unhealthy diet focus group expressed doubts about the scope for a EU-wide prescriptive approach in this area of health promotion, recommending regional level interventions instead. Overall, there seems to be untapped potential to couple cross-national health campaigns with legislative developments addressing life-style risks at the EU-level.

### Building the campaign coalition

A well-established strategy for ensuring an effective campaign is building strong partnerships with local actors and stakeholders, with the dissemination of messages aided by inter-agency collaboration [37]. This is especially crucial in cross-national campaigns in which EU institutions are the responsible party. Without strong local partners who are empowered to take ownership of elements of a campaign, international organisers run the risk of overlooking or bypassing certain contextual specificities. Furthermore, in order for local partners to work effectively they must be involved in the inception and planning phases to ensure that a campaign reflects local idiosyncrasies from the outset. In addition to their role as local ‘navigators’, local partners tend to have access to services, materials and human resources necessary for the implementation of a campaign.

Of particular relevance for the composition of a campaign coalition is the possible conflict of interest posed by including industry representatives as campaign partners, apart from the case of smoking where expert consensus calls for their exclusion. The food industry can be responsive to the need to take steps to address healthy diet [38] and the availability of healthy options in stores and supermarkets is an important determinant of healthy food consumption [33]. Conversely, advertising practices of the food and beverage industry are not necessarily aligned with the goals of healthy eating [39] and members of the industry are valuable partners only as long as the aims of campaigns are attuned to corporate practices and interests [38]. There is also some concern about the impact that industry lobbying has had on public health policies at the EU level [8].

The inherent diversity in cultural and institutional frameworks lead us to conclude that cross-national campaigns are most effective when the role of the EU as coordinating body is to provide a guiding campaign framework, or blueprint, that countries can adopt, while making the necessary adaptations and modifications to meet their specific needs, to take into consideration local political frameworks and socio-cultural traditions, and to take advantage of the resources available in their local context.

### Limitations of the study

We acknowledge two limitations of the current study. The first follows from its exploratory nature. As the research examines a relatively unexplored field, the challenges identified and the suggestions offered are neither exhaustive nor comprehensively defined. We encourage more detailed research into the specificities of cross-national health communication campaigns. The second limitation stems from the relatively limited sample of experts in our study. Further studies drawing on larger samples are encouraged to address this shortcoming.

## Conclusions

This study investigated the challenges in planning and operationalising cross-national health communication campaigns in Europe. Despite its European focus, the findings are instructive for cross-national communication campaigns for the prevention of chronic diseases carried out in other regional contexts. Gathering insight through expert focus groups organised around the four main risk factors for chronic diseases (smoking, alcohol consumption, sedentary lifestyle and unhealthy diet), it is evident that while cross-national campaigns have explicit advantages, they must address national and sub-national variations across a range of dimensions if they are to be successful. These include primarily epidemiological, cultural, and legislative differences. In order to do so, a keen awareness and focus on the local context is needed. This entails the active inclusion of local actors in the design, planning and implementation phases of campaigns. That said, deciding which roles and tasks should be decentralised and which should be centralised within higher levels of governance remains a delicate and balancing act. Further research is needed into transferability of know-how from local/national to cross-national settings, and into balancing standardised components of a cross-national campaign with locally adapted ones. A key caveat remains that isolated from legislative action, cross-national communication campaigns are limited in their potential to produce a long-term effect on behaviours and therefore health outcomes. We therefore encourage further studies to explore the two issues in conjunction.

## Supporting information

S1 Material Key design principles (KDPs) for a cross-border public health campaign. (DOCX)
