# Supplementary material for: It’s better together! European perspective on benefits and challenges associated with cross-border health communication campaigns
Source: PLoS One. 2018 Oct 17;13(10):e0204882. doi: 10.1371/journal.pone.0204882 (PMC6192590; doi:10.1371/journal.pone.0204882)
Supplement: S1 Material — (DOCX) [file pone.0204882.s001.docx]

**S1 Material. Key design principles (KDPs) for a cross-border public health campaign**

All material presented here has been reproduced from: DG Health and Food Safety (2015) Scoping study on communication to address and prevent chronic diseases [Internet]. Luxembourg; 2015. Available from: <https://ec.europa.eu/health//sites/health/files/major_chronic_diseases/docs/2015_chronic_scopingstudy_en.pdf>

We encourage the reader to consult the original source for further details on methodological aspects, the results and a discussion of their applicability and relevance.

**Summary of KDP**


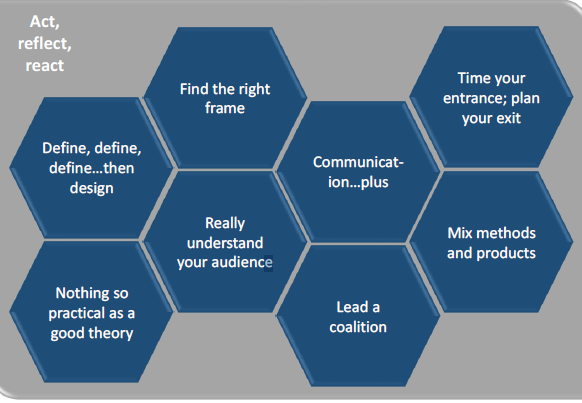


**Underpinning principle: act, reflect, react**

The use of evidence is fundamental to good campaigning. The exact role of evidence depends upon the stage of the campaign, as does the nature of the evidence needed. Yet throughout, campaigns should aim for a cycle of continuous improvement, rather than the design / implementation / evaluation of ‘the perfect campaign’. This requires an open mind set as well as strong technical skills.

**Define, define, define…then design**

You need a clear understanding of the nature, scale and evolution of the problem(s) to be addressed. Background research is the backbone of campaign design. This also includes defining the target group of interest and the behavioural outcome(s) desired with as much precision as possible.

**Nothing so practical as a good theory**

Accepting that no single model or discipline has ‘the answer’, it is important to draw behavioural insights from theory developed in, e.g., psychology, sociology, economics, history, marketing, etc. These can be used to set out the theory of change for the campaign: by what mechanisms will your intervention translate into changes in behaviour?

**Communication…plus**

Greater effects can be achieved by combining communications with other interventions, e.g. taxation, service provision, regulation, public engagement. In isolation, provision of information is unlikely to be sufficient to bring about widespread behavioural change.

**Mix methods and products**

Effective campaigns typically use more than one media type – using different means of communicating the same message. The choice and mix of channels always depends on the context, resources available and media used by the target group in the particular countries included in the scope of a campaign. The addition of ‘tools’ (e.g. self-assessment tools, progress trackers) to help promote the desired behaviour should also be considered; if provided digitally they can be spread widely and can create a connection with your target group that can be used again.

**Really understand your audience**

Formative research is vital in gaining insights into the motivations, values, attitudes, behaviours, influencers and media choices of the target group. This is especially important in cross-cultural settings and / or when targeting specific groups to address inequalities. Doing this groundwork early on will fundamentally shape your campaign strategy.

**Find the right frame**

The choice of messages should be the result of all the preliminary research and analysis. It very much depends on the context, objectives and target audiences of the campaign. Radically different approaches are available: from ‘shock tactics’ to positive testimony; from emphasising long- to short-term benefit. Effectiveness then depends upon the cultural context within which it is received.

**Lead a coalition**

How will your efforts be supported and multiplied? Which organisations can be used to transmit your message? How does this relate to the influencers of your target group? For partnerships to be effective and sustainable they need to constitute a win-win for all partners; they also require time and effort.

**Time your entrance; plan your exit**

A ‘life course’ approach will help with the definition of your target group by considering behaviours within the context of ‘their time of life’. There are points in life (e.g. first pregnancy) when individuals are more open to change. Thinking seasonally will also help to time the campaign within the year, e.g. using ‘points in the year’, such as New Year’s resolutions or summer holidays. At some point, your campaign will end. This point will be dictated by multiple factors, but this doesn’t affect the need to consider sustainability from the outset.
